# CLARIPED: a new tool for risk classification in pediatric emergencies

**DOI:** 10.1016/j.rppede.2016.02.002

**Published:** 2016

**Authors:** Maria Clara de Magalhães-Barbosa, Arnaldo Prata-Barbosa, Antonio José Ledo Alves da Cunha, Cláudia de Souza Lopes

**Affiliations:** a Instituto D’Or de Pesquisa e Ensino (Idor), Rio de Janeiro, RJ, Brazil; b Departamento de Pediatria, Faculdade de Medicina, Universidade Federal do Rio de Janeiro (UFRJ), Rio de Janeiro, RJ, Brazil; c Instituto de Medicina Social (IMS), Universidade do Estado do Rio de Janeiro (Uerj), Rio de Janeiro, RJ, Brazil

**Keywords:** Triage, Emergency medical services, Pediatrics

## Abstract

**Objective:**

To present a new pediatric risk classification tool, CLARIPED, and describe its development steps.

**Methods:**

Development steps: (i) first round of discussion among experts, first prototype; (ii) pre-test of reliability, 36 hypothetical cases; (iii) second round of discussion to perform adjustments; (iv) team training; (v) pre-test with patients in real time; (vi) third round of discussion to perform new adjustments; (vii) final pre-test of validity (20% of medical treatments in five days).

**Results:**

CLARIPED features five urgency categories: Red (Emergency), Orange (very urgent), Yellow (urgent), Green (little urgent) and Blue (not urgent). The first classification step includes the measurement of four vital signs (VIPE score); the second step consists in the urgency discrimination assessment. Each step results in assigning a color, selecting the most urgent one for the final classification. Each color corresponds to a maximum waiting time for medical care and referral to the most appropriate physical area for the patient's clinical condition. The interobserver agreement was substantial (kappa=0.79) and the final pre-test, with 82 medical treatments, showed good correlation between the proportion of patients in each urgency category and the number of used resources (*p*<0.001).

**Conclusions:**

CLARIPED is an objective and easy-to-use tool for simple risk classification, of which pre-tests suggest good reliability and validity. Larger-scale studies on its validity and reliability in different health contexts are ongoing and can contribute to the implementation of a nationwide pediatric risk classification system.

## Resumo

**Objetivo:**

Apresentar um novo instrumento de classificação de risco pediátrico, o CLARIPED, e descrever as etapas de seu desenvolvimento.

**Métodos:**

Etapas do desenvolvimento: (i) primeira rodada de discussão entre especialistas, primeiro protótipo; (ii) pré-teste de confiabilidade, 36 casos hipotéticos; (iii) segunda rodada de discussão para ajustes; (iv) treinamento da equipe; (v) pré-teste com pacientes em tempo real; (vi) terceira rodada de discussão para novos ajustes; (vii) pré-teste final de validade (20% dos atendimentos de cinco dias).

**Resultados:**

O CLARIPED apresenta cinco categorias de urgência: Vermelha (emergência), Laranja (muito urgente), Amarela (urgente), Verde (pouco urgente) e Azul (sem urgência). A primeira etapa da classificação inclui a aferição de quatro sinais vitais (escore Vipe); a segunda etapa consiste na avaliação de discriminadores de urgência. Cada etapa resulta na atribuição de uma cor, seleciona-se a de maior urgência para a classificação final. Cada cor corresponde a um tempo máximo de espera pelo atendimento médico e ao encaminhamento à área física mais adequada à condição clínica do paciente. A concordância interobservador foi substancial (*kappa*=0,79) e o pré-teste final, com 82 atendimentos, evidenciou boa correlação entre a proporção de pacientes em cada categoria de urgência e o número de recursos usados (*p*<0,001).

**Conclusões:**

O CLARIPED é um instrumento para classificação de risco simples, objetivo e de fácil uso, cujos pré-testes sugerem boa confiabilidade e validade. Estudos em maior escala sobre sua validade e confiabilidade em diferentes contextos de saúde estão em curso e podem contribuir para a adoção de um sistema de classificação de risco pediátrico em âmbito nacional.

## Introduction

In the last two decades, a major challenge in health care has been to find solutions to the increased overcrowding in emergency service hospitals. One of the strategies adopted in many countries to deal with this problem was the implementation of triage systems used to classify each patient's degree of clinical urgency shortly after his/her arrival to the Emergency Department (ED), establishing a waiting list based on clinical risk, and not in order of arrival, to undergo medical evaluation and treatment.

The Australian Triage Scale (ATS), Canadian Triage & Acuity Scale (CTAS), Manchester Triage System (MTS), and Emergency Severity Index (ESI) are the tools for triage in emergency services most used worldwide, all with five levels of urgency.^1^^,^ 2

In Brazil, the risk classification system developed by the Ministry of Health in the Qualisus Program has only four emergency categories, does not address the pediatric group peculiarities, and has not achieved significant national adherence.^3^^,^ 4 On the other hand, those developed in Europe, North America, and Australia are complex, which hinders large-scale adoption in a heterogeneous health context as the Brazilian. Moreover, there are insufficient literature on the validity and reliability of the pediatric versions of these triage systems.

The aim of this study is to present a new risk classification tool, the CLARIPED, for pediatric emergencies and describe the steps of its development. The intent is to obtain a reliable and valid tool that is best suited to the Brazilian health context.

## Method

The development of the CLARIPED tool was performed in seven steps: (i) meetings of experts to discuss the new instrument up to the proposal of a prototype (first half 2013); (ii) first pre-test, with the prototype application in 36 hypothetical cases, submitted to 9 professionals of the emergency service after 3h of training (August 2013) and evaluation of the agreement among them (kappa-statistic measure); (iii) new round of discussions on the results of the first pre-test, which yielded changes in the prototype; (iv) new training of triage professionals, with supervision and discussion of real cases by a specialist (September 2013); (v) second pre-test performed in real time with the participation of all triage team, using the second prototype after obtaining written informed consent from all guardians (October and November 2013); (vi) new round of discussions and the final version presentation with the incorporation of the proposed modifications; (vii) final pre-test to evaluate the association between emergency categories and a proxy outcome of urgency (number of resources used); for such, the final CLARIPED version was applied retrospectively in a systematic sample of 20% of cases attended in five days of December 2013; urgency levels were compared with the number of diagnostic and/or therapeutic resources used; triage and clinical data were obtained through medical charts review. The study was approved by the Institutional Review Board of the Instituto D’Or de Pesquisa e Ensino (IDOR), under the number 209 075/2013.

## Results

A group of experts (three doctors and two nurses) with extensive experience in pediatric emergencies was assembled in order to choose and test a risk classification tool for the pediatric emergency department. After extensive literature review, it was concluded that the four triage systems designed in North America (CTAS and ESI), United Kingdom (MTS), and Australia (ATS) were not suitable for our country, as they are extensive, complex or lack specific-pediatric features. The South African Triage Scale (SATS),^5^ although simpler and more adaptable to the Brazilian health context, only had four levels of urgency, insufficient stratification of pediatric age groups, in addition to having few studies of its use in children. It was decided, therefore, to design a new triage system specific for pediatric emergencies.

### The CLARIPED system

CLARIPED comprises five urgency categories: Red (imminent life threat), Orange (very urgent), Yellow (urgent), Green (little urgent) and Blue (not urgent). Each category is assigned a maximum waiting time for medical evaluation and referral to an appropriate service area of adequate care to the patient's level of urgency, as follows: *red*, immediate care in the ressucitation room; *orange*, care within 10min in the observation room; *yellow*, up to 30min, waiting room; *green*, up to 90min, waiting room; and *blue*, up to 180min, waiting room. The risk classification should start in a maximum of 10min after patient's arrival and registration; it should be performed by a nurse and last 2-5min.

The first step starts with six questions about complaints, drug allergies, regular pediatric care, associated morbidities, using medications and last measured weight. Next is the evaluation of four vital signs: respiratory rate (RR), heart rate (HR), oxygen saturation (SpO_2_), and skin temperature (Temp). Each vital sign measured is assigned a value from 0 to 4, which will compose the Pediatric Vital Signals (VIPE) score that ranges from 0 to 12, consisting of the sum of the first three parameters values subtracted from the temperature value in case of increased HR (RR+HR+SpO_2_-Temp, if increased HR). The VIPE score should then be associated with one of five colors: score 0=Blue; 1-2=Green; 3-5=Yellow; 6-9=Orange; ≥10=Red (Table 1).

**Table 1:** VIPE score calculation (vital signs in Pediatrics).

*Newborn to 2 months old*							
	4	2	1	0	2	3	4
RR	<16	16-19	20-29	30-60	61-80	81-90	>90
HR	<81	81-90	91-110	111-149	150-179	180-189	>189
SpO_2_	<90	90-92	93-94	95-100			

*3 months to 11 months old*							
	4	2	1	0	2	3	4
RR	<16	16-19	20-24	25-50	51-70	71-80	>80
HR	<71	71-80	81-100	101-139	140-169	170-179	>179
SpO_2_	<90	90-92	93-94	95-100			

*1 year to 4 years old*							
	4	2	1	0	2	3	4
RR	<13	13-15	16-19	20-40	41-60	61-70	>70
HR	<61	61-70	71-90	91-119	120-149	150-169	>169
SpO_2_	<90	90-92	93-94	95-100			

*5 years to 12 years old*							
	4	2	1	0	2	3	4
RR	<11	11-14	15-17	18-24	25-36	37-50	>50
HR	<51	50-60	61-70	71-109	110-129	130-149	>149
SpO_2_	<90	90-92	93-94	95-100			

*>12 years*							
	4	2	1	0	2	3	4
RR	<10	10	11	12-16	17-22	23-29	>29
HR	<41	41-50	51-60	61-99	100-119	120-139	>139
SpO_2_	<90	90-92	93-94	95-100			

*Assessment of axillary temperature (regardless of age)*							
*If HR is increased (score 2, 3 or 4) Subtract:*							
				0	-1	-2	
AxT				36-37.4	37.5-38.5	>38.5	

VIPE score (0-12) is the sum of the points assigned to each vital sign. However, if heart rate is increased (score 2, 3 or 4);-1 should be subtracted from the final score if the axillary temperature is between 37.5 and 38.5ºC and-2 if the axillary temperature (AxT) is>38.5ºC.Urgency classification: Blue (0), Green (1-2), Yellow (3-5), Orange (6-9), and Red (≥10).

The second step is to consult the tables of discriminators categorized by type or organ system involved (lines) and by urgency levels (columns). Assessment of five general discriminators (pain, general appearance, fever report, age, and return to the ED) is mandatory for all patients (Table 2). The other discriminators are evaluated according to the patient's complaint (Table 3). It is not necessary to consult all lines and columns of the second table, only the relevant lines associated with the reported complaints and the columns corresponding to levels of urgency higher than the assigned by the VIPE score. If there is a discriminator corresponding to a higher level of urgency than that assigned by the VIPE score, the higher level of urgency will determine the final classification. The urgency determined by the VIPE score may not be decreased, only increased from the evaluation of the discriminators.

**Table 2:** CLARIPED general and mandatory discriminators.

Discriminator	Red	Orange	Yellow	Green	Blue
Maximum waiting time	Immediate	10min	30min	90min	180min
General appearance	Critical look	Looks very ill	Looks ill	Looks little ill	Very good
		Important prostration	Mild to moderate prostration	No prostration	Does not look sick
Pain (level)^a^		Strong (7-10)	Moderate (4-6)	Mild (1-3)	No pain (0)
Fever (axillary temperature)^b^		Fever report ≥38.5ºC in<3 months	Fever report ≥37.5ºC in<3 months old		
			Fever report ≥38.5ºC in<3 years old		
			Fever report ≥39.5ºC at any age		
Age			Newborn (≤28 days)		
Return			1 return in<24h		
			2 returns in<72h		

a The level of pain should be evaluated by appropriate scales for the patient's age, such as FLACC Scale or Faces Scale (<5 years) and Visual Analogic Scale (>5 years).

b Fever report (axillary temperature-AxT) measured with thermometer in the current disease (any peak in the last 24h).

**Table 3:** CLARIPED discriminators according to reported complaints.

Discriminator	Red	Orange	Yellow	Green	Blue
Level of consciousness	Unconscious (coma)	Altered (drowsiness and stupor)			
Airway/breathing	Cyanotic lips	Dyspnea^a^	Tachypnea^b^	Coryza and/or sneezing	
Apnea	Stridor^c^	Hoarseness^d^ in<2 years	Cough		
Cardiovascular	Absent pulses	Weak pulses			
Cyanotic extremities	Capillary refill>2s.				
Neurological	Seizure at the time of care	Acute focal deficit	Seizure in the past 12h		
	Postictal state	CBG:^e^ 40-60 (≤1 year) or 60-80 (>1 year)			
	CBG:^e^<40 (≤1 year) or<60 (>1 year)				
Gastrointestinal and genitourinary		Signs of dehydration^f^	Current seizure or report of persistent vomiting	History of vomiting and/or diarrhea in the past 72h	
	Urinary retention with palpable bladder	>5 bowel movements/day or bloody diarrhea			
	Scrotal pain and/or swelling	Current and persistent abdominal pain			
		Intermittent abdominal pain<2 years			
Trauma	Polytrauma^g^	TBI with report of loss of consciousness and/or vomiting	TBI with<12h and no report of loss of consciousness or vomiting	TBI with>12h without loss of consciousness and without vomiting	
	Open fracture or deformity				
	High energy mechanism^h^	Suspected fracture or limb dislocation			
Bleeding or wound^i^	Uncontrolled bleeding	Profuse bleeding	Controlled moderate bleeding	Small bleeding	Minor injury without bleeding
	Extensive injury	Moderate injury	Small wound with mild bleeding		
Burn^j^	Face and/or inhalation	Moderate>20%	Mild<10%		
	Electrical or circumferential or chemical burn				
Exogenous intoxication		Present (reported)			
Skin	Urticaria^l^ with stridor and significant respiratory difficulty	Urticaria^l^ with face edema	Extensive urticaria^l^	Rash without petechiae	Impetigo or local abscess
	Purpura^k^	Edema without hypertension	Local urticaria		
	Edema+BP>140×90	Signs of cellulite			
Locomotor			Claudication+fever Acute gait disorder		
Foreign body			Present		
Unspecific		Unable to stand	Inconsolable crying		
	Behavior change	Irritability			
		Suspected abuse			
Comorbidities	Diabetes, with severe hypoglycemia (CBG<20)^e^	Diabetes with hypo- or hyperglycemia: CBG^e^<60 or>400	Diabetes with CBG^e^ between 250 and 400		
	Immunosuppression with fever	Neuromuscular diseases^m^			
		Immunosuppression^n^ without fever			
Suspected dengue (always measure BP^o^ lying and sitting)	Signs of shock	Presence of warning signs^p^	Absence of warning signs^p^		
Signs of respiratory failure					

a Dyspnea: respiratory distress with presence of intercostal retractions.

b Tachypnea: increased RR according to the VIPE table (RR score>0).

c Stridor: noise during inspiration with varying degrees of respiratory distress.

d Hoarseness: hoarse cry or voice or cough, without stridor or respiratory distress.

e Capillary blood glucose (CBG mg/dL): perform the test in all patients with altered level of consciousness, recent or current seizure, lethargy or previous history of diabetes.

f Signs of dehydration: dry mouth, sunken eyes, not urinating in the last 12h, decreased skin turgor.

g Polytrauma: patients with traumatic injury in two or more organs.

h High energy mechanism (in the absence of information, consider any traffic accident as high energy mechanism): (a) motor vehicle accident>60km/h (belt collision); >40km/h (unbelted collision); >30km/h (motorcycle), and >10km/h (run over); (b) a fall from higher than 1m.

i Wound: (a) mild: abrasions and contusions requiring no suture; (b) moderate: contusions requiring sutures, but with controlled bleeding; (c) severe: extensive contusions requiring sutures, profuse and continuous bleeding.

j Burn: Rule of nines.

k Purpura: presence of petechia and/or ecchymosis.

l Urticaria: unlike other skin rashes; hyperemia and elevated plaques, usually very itchy, but not always.

m Neuromuscular diseases: chronic encephalopathy, myopathy, myelomeningocele, hydrocephalus.

n Immunosuppression: after chemotherapy, nephrotic syndrome in activity, chronic use of corticosteroids.

o Blood pressure (BP): Always measure in case of altered level of consciousness, recent or current seizure, suspected dengue fever, edema of the lower limbs or generalized (anasarca).

p Warning signs in dengue (presence of one or more signs): abdominal pain, persistent vomiting, respiratory distress, postural hypotension or dizziness, drowsiness, and/or irritability, spontaneous bleeding, decreased urine output, sudden drop in temperature, hypothermia, edema.

In the presence of discriminators indicating risk of life threatening, such ass seizures, impaired level of consciousness, apnea, cyanosis, and others, the patient must be sent for rapid or immediate medical care before any administrative procedure. The risk classification process is then performed retrospectively.

### Pre-test studies

The hypothetical clinical scenarios designed by experts for training had the following distribution of urgency levels: Red 11%, Orange 42%, Yellow 31%, Green 8%, and Blue 8%. The overall kappa for multiple observers resulting from the first pre-test with 36 hypothetical clinical scenarios was 0.79 and kappa for each urgency category was 0.93 (Red); 0.82 (Orange); 0.73 (Yellow); 0.65 (Green), and 0.93 (Blue), with a standard deviation of 0.03 and *p*<0.001, which represented substantial reliability.

The second pre-test, performed with patients in real time, determined changes for the treshholds and values attributed to the physiological parameters and for some discriminators (newborn, return in less than 24h, fever report, abdominal pain, and cranial trauma).

The final validity pre-test, included the retrospective analysis of 95 medical charts, selected by a systematic sampling of 20% of the cases attended in the first five days of December 2013. The aim was to evaluate the distribution of the levels of urgency and their association with an outcome, which could work as a proxy of urgency, such as the number of diagnostic and therapeutic resources used. The distribution of the levels of urgency was: Blue (4.2%); Green (34.7%); Yellow (41%); Orange (6.3%), and Red (0%). None of the attendances classified as Blue used a diagnostic and/or therapeutic resource in the emergency department; one third of the Green attendances, half of the Yellow ones used one resource and 20% used two or more resources; over 80% of the Orange ones used two or more resources (Table 4; *p*<0.001).

**Table 4:** Pilot pre-test of validity: use of treatment resources in accordance with the level of risk classification.

	Frequency *n* (%)	0 resource *n* (%)	1 resource *n* (%)	2 resources *n* (%)	≥3 resources *n* (%)	Total *n* (%)	*p*-value^a^
Blue	4 (4.2)	4 (100)	0	0	0	4 (4.9)	<0.001
Green	33 (34.8)	17 (51.5)	14 (42.4)	1 (3)	1 (3)	33 (40)	
Yellow	39 (41.0)	9 (23)	17 (43.6)	12 (30.8)	1 (2.6)	39 (47.6)	
Orange	6 (6.3)	0	1 (16.6)	2 (33.3)	3 (50)	6 (7.3)	
Red	0	0	0	0	0	0	
Missing	13 (13.7)						
Total *n* (%)		30 (36.6)	32 (39)	15 (18.3)	5 (6)	82 (100)	

a Chi-square test.

## Discussion

The proposal to develop a simple, objective, and easy to use tool for risk classification in pediatric emergencies, appropriate to a Brazilian health context, resulted in the CLARIPED. Brazil's continental magnitude and heterogeneity make it difficult to adopt a risk classification system nationwide. The option to develop a new instrument, instead of using other triage systems already evaluated, is justified. The most commonly used systems in the world were developed in countries in North America,^6^^,^ 7 Europe,^8^ and Australia.^9^ The health context of these countries is quite different from that of Latin America, both in terms of the epidemiology of health events and human resource training and availability. SATS, a simplified triage system, developed in an African country with many socioeconomic and demographic similarities with Brazil, could be a more appropriate instrument. It was developed and implemented in Cape Town in 2006, as Cape Town Score, and later adopted throughout the country as South African Triage Scale (SATS).^10^^-^ 12 CLARIPED kept the two-steps logistics of the SATS triage process, consisting of mesurement of physiological parameters, followed by assessment of discriminators. However, several modifications were performed in both steps and are detailed below.

Although SATS has five colors, strictly speaking it has only four levels of urgency, as the Blue level does not refer to non-urgent patients, but to patients who are dead on arrival. In the Brazilian health context, there is a large influx of outpatients to the emergency services.^3^^,^ 4 Thus, the existence of a level for non-urgent patients is justified, similar to other systems such as the MTS,^8^ ESI-4,^6^ PaedCTAS,^7^ and ATS.^9^ The triage tools with five emergency categories showed higher reliability and validity than tools with fewer categories.^13^

In CLARIPED, we opted for the stratification into five age groups, instead of the three groups used by SATS. This option is in agreement with the vital signs tables recommended by pediatric textbooks^14^^,^ 15 and other triage instruments that use four or more age groups.^6^^,^ 7 However, there is a lack of studies that validate the stratification of age groups for vital signs in Pediatrics. The Bedside Pediatric Early Warning System Score-Bedside PEWS Score), a tool designed to detect early clinical deterioration of hospitalized children, was derived from statistical methods and recently validated.^16^ The stratification into five age groups of CLARIPED was based on Bedside PEWS Score.

Although there is not yet an international consensus on the parameters to be included in a triage tool, there is evidence that vital signs should be an integral part of a safety risk classification process, particularly in children.^5^^,^ 17 ^,^ 18 However, vital sign measurements can be extremely difficult in emergency scenarios, particularly in small or uncooperative children. Selecting the most objective parameters that represent an effective contribution to the discrimination of emergency is crucial to achieve the goals of time and process improvement. In SATS, the first step includes evaluation of seven parameters: RR, HR, systolic blood pressure (SBP), Temp, level of consciousness, mobility, and presence of trauma. The pediatric revised version of SATS excluded the SBP assessment. The PaedCTAS^7^ recommends the measurement of HR, RR, and SpO^2^ as first order modifiers, while the ESI-4^6^ recommends the measurement of the same three vital signs only in certain circumstances. The ATS^19^ leaves at the discretion of the triage professional the need to measure vital signs. In CLARIPED, the four selected vital signs and the score assigned to each of them are based on studies addressing the development of two instruments that used statistical methods to assess the ability of some physiological parameters to predict clinical outcomes: the Bedside Pews^16^ and Pediatric Emergency Assessment Tool (PEAT),^20^ a multivariate model to predict three levels of care required for pediatric patients in the emergency department.

The inclusion of SpO_2_ in VIPE score was based not only on use of this parameter in other validated instruments, such as the ESI-4,^6^ PaedCTAS,^7^ and Bedside Pews Score,^16^ but also on evidence that patients with low SpO_2_ usually have no increased respiratory rate and that its measurement may generate significant changes in the management of a number of patients attended at pediatric emergency services.^21^^,^ 22

The exclusion of blood pressure measurement from the VIPE score was based on evidence that the benefit of its mandatory assessment in pediatric patients in the emergency department is limited.^23^ On one hand, hypotension is a late sign of shock in children and, on the other hand, the triage of children with hypertension in the emergency department may result in high prevalence of false positives.^24^ Other triage tools, such as MTS,^8^ Paed CTAS,^7^ ESI-4,^6^ and ATS,^19^ do not include mandatory measurement of blood pressure, but only the initial assessment of clinical signs of shock, such as thin pulses, delayed capillary refill, sweating, and pallor.

HR corrected by the degree of fever is a unique aspect of CLARIPED and was based on the fact that tachycardia is one of the systemic inflammatory response syndrome (SIRS) and sepsis parameters.^25^ A recent study proposes a similar correction in the evaluation of children with acute infection.^26^ No other triage system considers this correction. The assessment of the degree of fever is further considered in the discriminators step, according to age and follows the guidelines of the American College of Emergency Physicians.^27^ However, unlike other triage systems, CLARIPED takes into account not only the current fever, but the report of fever in the last 24h.

In CLARIPED, the evaluation of patient's level of consciousness and presence of trauma were moved to the step of discriminators assessment. Instead of considering the involvement of the patient's level of consciousness into four categories (awake, response to pain, response to voice, and non-responsive), as recommended in SATS,^11^ CLARIPED considers that the presence of any impairment of consciousness places the patient at the Orange level (very urgent). This strategy is already used in MTS,^8^ ESI-4,^6^ PaedCTAS,^7^ and ATS.^19^

In CLARIPED, trauma is assessed not only for its presence or absence, as recommended in SATS, but according to the aspects of mechanism, extension, location and presence of symptoms. The mechanism and trauma severity assessment is also used in other instruments. PaedCTAS^7^ and ATS^19^ assess the presence of a high energy mechanism, regardless of clinical symptoms, to classify patients into higher urgency categories. In MTS,^8^ there is a specific flowchart for major trauma in which patients are classified in emergency levels one, two, or at least three, depending on clinical manifestations.

Finally, the table of discriminators used in the second step of CLARIPED is very different from that used in SATS, in terms of content and organization. As in PaedCTAS,^7^ discriminators are grouped by organ systems. The Canadian tool, however, is much more extensive and complex, covering 167 complaints grouped into 17 organ systems.

A recent study of the SATS^5^ demonstrated that in pediatric triage the combination of the two steps, the evaluation of physiological parameters followed by the assessment of clinical discriminators, increased sensitivity (91%), compared with the use of each step alone (57.1% and 75.6%, respectively). In CLARIPED, it is considered that the VIPE score calculation has a lower weight in the tool's sensitivity, although this hypothesis has not been tested yet.

The final pre-test with a sample of 82 attendances found a distribution of emergencies similar to other studies.^28^^-^ 30 The association between the five levels of urgency and resource utilization suggests that the tool has good validity.

Some limitations may be identified in the development of the CLARIPED system. The first refers to the methodology for consensus on the proposed modifications and the choice and organization of discriminators. A Delphi method assuring the participating experts anonymity, interaction with controlled feedback, and statistical analysis of responses to successive rounds of interaction would be the preferred method to minimize bias and noise and ensure the prevalence of the majority opinion. On the other hand, the tool development process in several steps ensured the process systematization and a broad participation of the ED professionals in the development and improvement of the instrument up to its last version. The second limitation relates to the use of CLARIPED in other health care settings. It can be argued that the specific context in which the CLARIPED was conceived limits its application elsewhere. More specific discriminators related to ophthalmological or psychiatric emergencies, for example, are absent in CLARIPED. However, the search for the simplicity, objectivity, and training facility of the instrument can make its adaptation and dissemination viable in other less developed Brazilian regions by including more comprehensive and/or specific discriminators suitable to different settings. The concern in refining the discrimination between intermediate levels of urgency (Yellow [urgent] vs. Green [little urgent]) also makes the tool theoretically applicable to intermediate and low urgency care services. However the instrument is not suitable for pre-hospital triage during critical mass events. Specific instruments focusing on triage of critically ill patients in non-hospital settings are needed for this purpose.

It can be concluded that the development of CLARIPED for risk classification in pediatric emergencies resulted in a simple, objective, and easy to use tool, whose pre-tests suggest a good reliability and validity. Larger-scale studies of its validity, reliability, and application in different health contexts are underway and may contribute to the use of a pediatric risk classification system nationwide.
